# Beyond rhetoric in the pandemic treaty: Prioritizing equity and inclusion for marginalized populations

**DOI:** 10.1002/puh2.115

**Published:** 2023-08-15

**Authors:** Yusuff Adebayo Adebisi, Nelson Aghogho Evaborhene, Isaac Olushola Ogunkola, Calista Oluebube Onyike

**Affiliations:** ^1^ Nuffield Department of Population Health University of Oxford Oxford Oxfordshire UK; ^2^ Department of Vaccinology Faculty of Health Sciences University of the Witwatersrand, Johannesburg Johannesburg Gauteng South Africa; ^3^ Department of Public Health University of Calabar, Calabar Cross River Nigeria; ^4^ Faculty of Pharmaceutical Sciences University of Nigeria, Nsukka Nsukka Enugu Nigeria

Dear Editor,

The COVID‐19 pandemic has starkly exposed deep‐rooted inequities and vulnerabilities in our global health systems. These deficiencies disproportionately affect marginalized populations, such as racial and ethnic minorities, key populations of HIV, indigenous communities, people with disabilities and economically disadvantaged individuals [1, 2, 3]. An accord by the World Health Organization (WHO), known as the ‘pandemic treaty’ or WHO CA+, seeks to address these deficiencies. This treaty is currently under development by the Intergovernmental Negotiating Body (INB) [4]. In response to the international community's perceived failure in demonstrating solidarity and equity during the pandemic, the treaty's Zero Draft is being prepared as a comprehensive strategy for future pandemic prevention, preparedness and response [4]. As it stands, negotiations are ongoing, with the new instrument expected to be adopted at the World Health Assembly in May 2024 [4]. Despite the draft acknowledging the importance of addressing the needs of underserved communities, it is vital that actions match rhetoric through the active involvement of these communities in the process [4]. The rights and needs of vulnerable populations must be an integral part of the process throughout all stages. Notably, equity considerations, as highlighted by several advocates, seem to be insufficiently addressed in the current Zero Draft and need to be better incorporated [5].

The COVID‐19 pandemic has resulted in higher infection, hospitalization and mortality rates in minority communities, compared to the general population [6]. Barriers to healthcare access and other vital resources for vulnerable communities have been further amplified due to the pandemic. In the United States, for example, Black, Hispanic and Native American individuals are more likely to contract COVID‐19 and suffer severe outcomes, a disparity attributed to systemic racism, poor access to healthcare and a higher prevalence of underlying health conditions among these communities [7]. In the early days of the pandemic, the urban slums have seen more COVID‐19 infections than non‐slum areas in some settings, due to factors like overcrowding, limited healthcare and sanitation access and difficulties in physical distancing practices [8]. An apt illustration of global health inequity is seen in the distribution of COVID‐19 vaccines, where wealthy nations have hoarded the lion's share, leaving poorer countries struggling for access. Within these disadvantaged nations, marginalized communities, including ethnic and racial minorities, are further isolated, frequently facing barriers to accessing these crucial vaccines [9]. In Africa, the COVID‐19 pandemic has exacerbated existing inequities experienced by marginalized groups in under‐resourced areas, compounded by poor housing, limited healthcare access and inadequate water and sanitary facilities [3]. A critical step towards addressing these disparities is to protect these communities from further marginalization in global health initiatives [10]. One such way is through resource allocation towards research that collaborates with these communities, a step crucial not only in addressing the current pandemic but also in building a more robust and equitable global health infrastructure for future pandemics. This research should investigate social determinants of health, healthcare access and the effectiveness of culturally appropriate interventions.

Furthermore, the treaty should support community‐based participatory research and interdisciplinary approaches. For the successful inclusion of marginalized groups in the treaty's development and implementation, engagement with community leaders and advocates is essential. Their viewpoints and recommendations must be considered, and their feedback integrated into the treaty's framework. Even though the Zero Draft is yet to be finalized, it is essential to secure representation for marginalized communities in decision‐making entities and governance structures, with their perspectives and priorities consistently embedded in the decision‐making processes. Successful community engagement can involve advisory boards, including representatives from vulnerable communities, local non‐governmental organizations and healthcare professionals. This approach facilitates dialogue, gathers feedback and collaboratively develops strategies for addressing health disparities and ensuring equitable healthcare access.

The pandemic treaty presents an important milestone in global health cooperation, but it must actively prioritize the needs of underserved populations. Active engagement, representation in decision‐making bodies and investing in targeted research are key. Collaborations with local organizations and grassroots movements can provide valuable insights and tailored solutions to address unique needs of vulnerable communities. In doing so, we can create a more resilient and equitable global health landscape that leaves no one behind. Transcending boundaries of race, ethnicity, socio‐economic status and geography, we can pave the way for a future where health equity is a reality for all. We call on the INB to reassess the treaty drafting process promptly, to ensure meaningful participation by all stakeholders, especially those disproportionately affected due to uneven power dynamics.

## AUTHOR CONTRIBUTIONS

This paper was conceptualized by Yusuff Adebayo Adebisi. Yusuff Adebayo Adebisi, Nelson Aghogho Evaborhene, Isaac Olushola Ogunkola and Calista Oluebube Onyike were involved in the drafting of the work and revising it critically for important intellectual content.

## CONFLICT OF INTEREST STATEMENT

Yusuff Adebayo Adebisi and Isaac Olushola Ogunkola are Editorial Board members of Public Health Challenges and co‐authors of this article. To minimize bias, they were excluded from all editorial decision‐making related to the acceptance of this article for publication.

## FUNDING INFORMATION

This paper did not receive any specific grant from funding agencies in the public, commercial or not‐for‐profit sectors.
